# MiRNA expression profiling and clinical implications in prostate cancer across various stages

**DOI:** 10.1038/s41598-025-92091-9

**Published:** 2025-03-05

**Authors:** Zhengjie Xiang, Tao Lin, Jian Ling, Zuhuan Xu, Ruizhen Huang, Honglin Hu

**Affiliations:** Department of Urology, The Second Affiliated Hospital of Nanchang University, No.1 Minde Road, Nanchang, 330006 Jiangxi People’s Republic of China

**Keywords:** Prostate cancer, MicroRNAs, High-throughput sequencing, Prostate, High-throughput screening

## Abstract

This study aims to screen and identify microRNA (miRNA) expression profiles across different stages of prostate cancer (PCa) and benign prostatic hyperplasia (BPH) using high-throughput sequencing. The study seeks to determine whether specific miRNAs show consistent differential expression across various stages of PCa, with the goal of identifying potential biomarkers relevant to disease progression. In this study, a total of 12 specimens of PCa and BPH were collected from September 2021 to June 2022 in the Second Affiliated Hospital of Nanchang University, 330,006, P.R. China (including 3 specimens of early localized tumor, local invasion, and distant metastasis tumor and 3 specimens of BPH). The expression profile of miRNA was screened by high-throughput sequencing technology, and the differentially expressed miRNA between each group was screened by relevant bioinformatics analysis. Further targeted miRNA site analysis GO enrichment analysis and KEGG enrichment analysis of miRNA-derived genes were performed on the above differentially expressed miRNAs. Finally, the expression of hsa-miR-6715b-3p in PCa tissues was verified using qRT-PCR assay. A total of 1526 miRNAs were identified through high-throughput sequencing. By comparing groups, 228 differentially expressed miRNAs were identified, with 100 upregulated and 128 downregulated. Additionally, 69 novel miRNAs were predicted. qRT-PCR results showed that hsa-miR-6715b-3p was highly expressed in PCa tissues compared to BPH tissues. This study presents a preliminary investigation of the miRNA expression profiles in PCa and identifies hsa-miR-6715b-3p as a promising biomarker for disease progression. Our findings validate the high expression of hsa-miR-6715b-3p in PCa tissues and highlight its potential role in critical oncogenic pathways. These results provide a theoretical foundation for further functional studies to explore its clinical utility and its role in therapy resistance and disease progression, contributing to the growing knowledge of miRNA-based biomarkers in PCa.

**Supplementary Information:**

The online version contains supplementary material available at 10.1038/s41598-025-92091-9.

## Introduction

PCa has become the most prevalent and harmful malignancy of the male genitourinary system worldwide, posing a serious threat to men’s health. Its incidence and mortality are very high, especially among older men. With 1,466,718 new PCa cases worldwide, PCa ranks fourth after lung, breast and colorectal cancers, and with 396,773 deaths, PCa ranks eighth in mortality^1,2^. Although the incidence of PCa in China is relatively low compared to Western countries, rapid economic development, increased life expectancy, and the widespread adoption of early screening methods have contributed to a significant rise in PCa incidence and mortality. The National Cancer Centre of China has released an update on the burden of malignant neoplasms in China in 2022, in which PCa has a total of 134,200 cases, ranking ninth among all cancers in the country. The incidence rate of PCa is 9.68 (1/10^5^), ranking sixth among male malignant tumours. 47,500 deaths with a mortality rate of 6.59 (1/10^5^), ranking seventh among male malignant tumours^3,4^. At present, about 85% of men newly diagnosed with PCa show localized early tumors. Although conventional prostate-specific antigen (PSA) testing has significantly improved early detection, the medical and scientific community is still debating its benefits, because there is no consensus on whether it can effectively reduce the risk of death from the disease^5^. Patients in the PSA gray zone face significant diagnostic challenges, as mildly elevated PSA levels do not necessarily indicate PCa, and excessively high levels may be associated with benign conditions such as BPH^6^. Some studies suggest that patients in the PSA gray zone may exhibit more complex clinical features, and therefore, the development of new biomarkers, especially miRNAs, could help improve the diagnostic accuracy in the PSA gray zone and provide more sensitive and specific tools for early prostate cancer screening^7,8^. Due to the molecular heterogeneity of PCa, the identification of specific molecular markers through the REMARK guidelines is a reasonable approach to accelerate the diagnosis and treatment of PCa, paving the way for personalized medicine^9^. We hypothesize that specific miRNAs are consistently dysregulated across multiple stages of PCa and may serve as potential biomarkers for disease progression. To test this hypothesis, we conducted high-throughput sequencing to compare miRNA expression profiles in early localized, locally invasive, and metastatic PCa, as well as BPH tissues. Our goal was to identify common differentially expressed miRNAs across all PCa stages and validate their expression in independent patient samples. By demonstrating that hsa-miR-6715b-3p is significantly overexpressed in PCa tissues, this study aims to establish a foundation for miRNA-based biomarkers and precision medicine in prostate cancer. With the development of protein and messenger RNA (mRNA) in different clinical application scenarios, the potential of miRNA as a biomarker of PCa has been paid more and more attention and is expected to play a greater role.

## Materials and methods

### Materials and reagents

1. The RNA Nano 6000 Assay Kit (Agilent Technologies, CA, USA).
2. RNase R (Epicentre, USA).
3. Ribo-zero™ rRNA Removal Kit (Epicentre, USA).
4. USER Enzyme (NEB, USA).
5. RNA Library Prep Kit for Illumina^®^ (NEB, USA).
6. TruSeq PE Cluster Kit v3-cBot-HS (Illumia).
7. AMPure XP system (Beckman Coulter, Beverly, USA).

### Instruments and equipment

1. NanoPhotometer^®^ spectrophotometer (IMPLEN, CA, USA).
2. Qubit^®^ 2.0 Flurometer (Life Technologies, CA, USA).
3. the Bioanalyzer 2100 system (Agilent Technologies, CA, USA).
4. Illumina Hiseq 4000 (illumina, USA).

### Preparation of samples

#### Experimental grouping

Specimens of PCa and BPH were collected, including 3 cases of early localized, locally invasive, and metastatic PCa, as well as 3 cases of BPH. All patients underwent laparoscopic radical prostatectomy between September 2021 and June 2022 in the Department of Urology of the Second Affiliated Hospital of Nanchang University. This study was approved by the Ethics Committee of the Second Affiliated Hospital of Nanchang University. Each patient gave written informed consent before participating in this study. All patients included in the study did not receive radiotherapy, endocrine therapy, and related biological agents, and excluded other primary tumor diseases and severe organ dysfunction such as heart, liver, and kidney. After postoperative pathological examination, we found that the patient had PCa and was treated according to the TNM staging criteria of the International Union against Cancer^6^. In this experiment, all selected PCa tissue specimens and BPH specimens were obtained in advance with the patient’s informed consent and signed the informed consent form. The clinical records of all patients were recorded.

In addition, prostate tissue specimens from 10 PCa and 10 BPH patients were collected to validate the differentially expressed miRNAs by real-time fluorescence quantitative PCR (qRT-PCR) detection. They were divided into two groups: experimental group: 10 PCa patients; control group: 10 BPH patients.

#### Collection of specimens

PCa tissues and BPH tissues were collected during surgery. The shorter the in vitro time of the specimen, the better, and the in vitro time should be controlled less than 20 min. After the specimen was obtained, it was quickly placed in the RNA protection solution, placed at 4 °C overnight, placed in a negative 80 °C refrigerator, and marked. PCa patient specimens: including prostate biopsy specimens and radical prostatectomy specimens. BPH specimens: including transurethral plasma kinetic resection of prostate specimens. The TNM staging information is shown in Table 1 below.

**Table 1:** Information on the 12 patients who underwent high-throughput sequencing.

Patient number	Age (years)	Prostate TMN staging
WXH	74	BPH
HQ	79	BPH
CYC	67	BPH
HLZ	66	T_2_N_0_M_0_
CAG	66	T_2_N_0_M_0_
LCY	64	T_2_N_0_M_0_
ZGA	70	T_3b_N_1_M_0_
PWJ	73	T_3a_N_0_M_0_
XW	70	T_3b_M_0_N_0_
QCL	63	T_4_N_0_M_1_
WJQ	64	T_4_N_0_M_0_
WWC	63	T_4_N_0_M_1_

### Experimental methodology

#### RNA extraction and quality control

Total RNA was extracted using the Trizol method. Agarose gel electrophoresis was employed to assess DNA contamination and RNA integrity, while RNA concentration and purity (OD260/280) were measured using a Nanodrop spectrophotometer. If the OD260/280 value range was 1.8–2.1, the purity of RNA was qualified.

#### Library construction

**Fig. 1:**
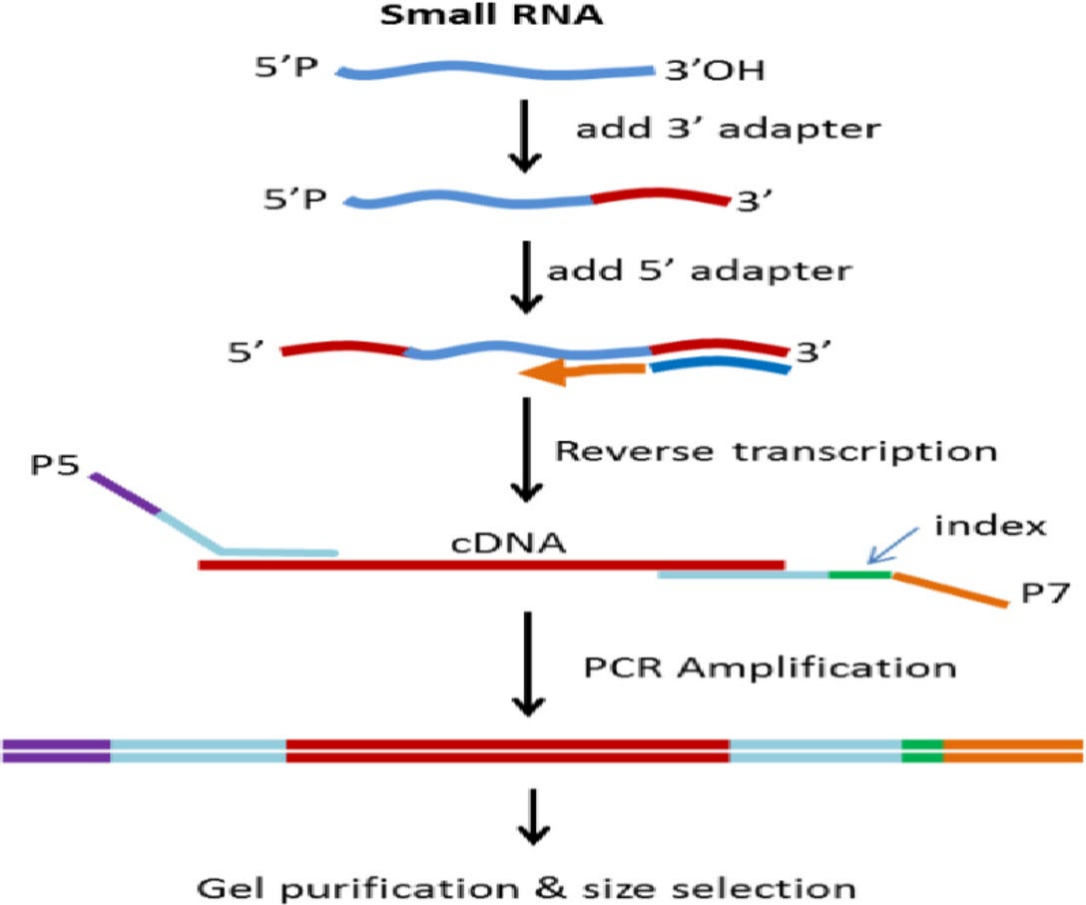
The principle diagram of cDNA library construction.

First, we used a high-sensitivity Agilent 2100 kit to accurately assess the RNA integrity. After rigorous sample testing, we constructed the library using the Small RNA Sample Pre Kit, taking advantage of the unique structural features of Small RNAs (with a complete phosphate group at the 5’ end and a hydroxyl group at the 3’ end). Using total RNA as the starting material, we directly ligated adapters to both ends of the Small RNA, followed by reverse transcription to synthesize cDNA. It is essential to ensure the purity and integrity of the extracted cDNA to avoid contamination and degradation of the DNA, which could interfere with subsequent library preparation and sequencing results. The cDNA was then amplified by PCR, and target DNA fragments were separated by PAGE gel electrophoresis. The resulting bands were gel-extracted to obtain the cDNA library (Fig. 1).

#### Library quality testing

After library construction, the concentration is initially quantified using the Qubit 2.0 and diluted to 1 ng/µl. The library insert size is assessed using the Agilent 2100 Bioanalyzer, with the insert size distribution ranging from 250 to 300 bp. Once the insert size meets expectations, qPCR is performed for precise quantification of the library’s effective concentration, which is > 2 nM, ensuring the quality of the library.

#### Sequencing

After the libraries were qualified, the samples were pooled according to the effective concentrations of different libraries and standards, and then sequenced using the Illumina SE50 platform. The constructed RNA sequencing library was then loaded into the sequencer for sequencing to generate raw sequencing data.Sequencing of RNA samples was performed by Shanghai Jikai Gene Technology Co. on the Illumina Hiseq4000 (Illumina, USA) platform.

#### Bioinformatic analysis

Firstly, the quality of sequencing data is evaluated, including data volume, alignment rate, and sequencing error rate. The main process is to remove low-quality data and obtain high-quality and standard-compliant data, which is called ‘clean reads ‘. The tags of rRNA, scRNA, snoRNA, snRNA, tRNA, and other small RNAs were removed by comparison with Genebank, Rfam, and other databases. The existing miRNAs were identified by the miRBase database, and then new miRNAs were predicted by using miREvo and mirdeep2 software. Finally, we obtained the target miRNAs of AURKB from two prediction databases, including miRDB (http://mirdb.org/miRDB/) and TargetScan (https://www.targetscan.org/vert_72/). We then retrieved the common target miRNAs from these two databases and ultimately constructed a network graph of AURKB with the target miRNAs.

The clean sequencing data are compared with the reference genome to determine the origin and localisation of RNA sequences. The high-throughput sequencing matrix is obtained by calculating the coverage and number of RNA sequences. By normalising the matrix data in TPM format and applying the DEGseq 2.0 package of R software, we can effectively detect and identify expression differences in miRNAs. Screening criteria: |Fold change (FC)|>2, *p* < 0.05. To better understand the functional significance of these differentially expressed miRNAs, we performed Gene Ontology (GO) and Kyoto Encyclopedia of Genes and Genomes (KEGG) analyses. Enrichment pathways with corrected p-values < 0.05 are significant enrichment results.

## Results

### The result of MiRNA identification and differential MiRNA screening

**Fig. 2:**
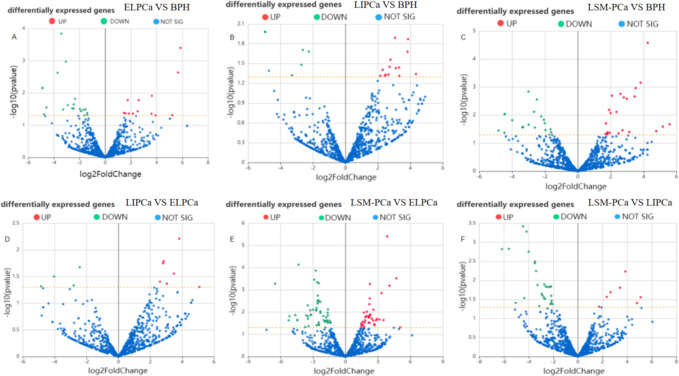
Volcano map of differentially expressed miRNAs in prostate cancer. (early localized PCa, ELPCa; local invasion PCa, LIPCa; late-stage metastasis PCa, LSM-PCa; Benign prostatic hyperplasia, BPH; Prostate cancer, PCa).

**Fig. 3:**
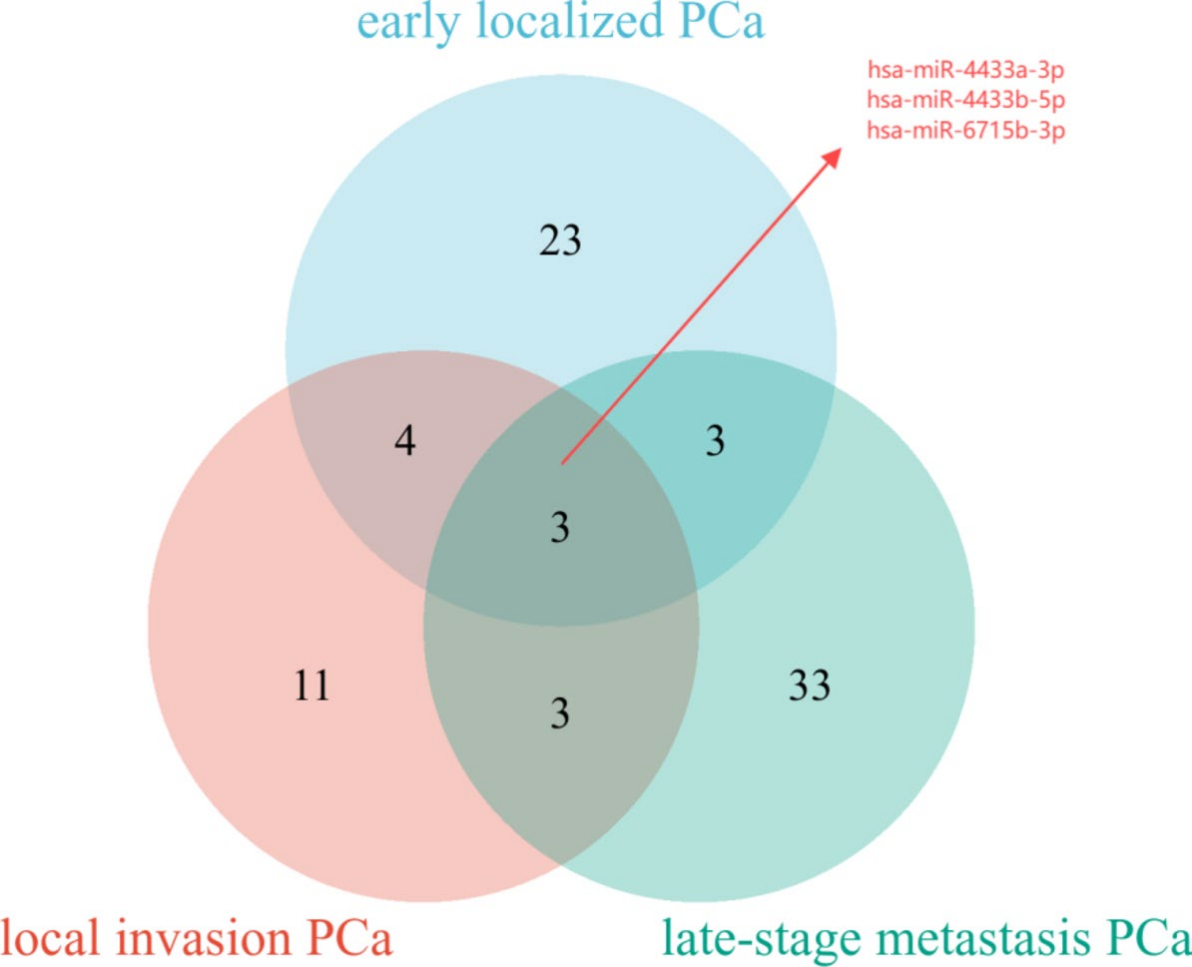
The venn diagram of differentially expressed miRNAs in different stages of prostate cancer (Prostate cancer, PCa).

Firstly, we detected a total of 1526 miRNAs using high-throughput sequencing, and identified 1457 known miRNAs using these tools such as the miRBase database, miREvo, and mirdeep2 software. By using DEGseq software, we can effectively detect and identify the expression level of miRNAs, and can effectively identify miRNAs with significantly different expression levels in the case of |Fold change (FC)|>2, *p* < 0.05, to facilitate our follow-up study. Through the comparison between each group, we found that a total of 33 differentially expressed miRNAs were identified in early invasive PCa, 21 differentially expressed miRNAs were identified in locally invasive PCa, and 42 differentially expressed miRNAs were identified in advanced metastatic PCa compared to BPH (Supplementary Material Table 1). The differential miRNAs between different stages of PCa are shown in the Supplementary Material Table 1. The results were visualised using volcano plots (Fig. 2), Differentially expressed miRNAs were identified using |Fold change (FC)| >2 (equivalent to log2FC > 1 or < − 1) and *p* < 0.05. For clarity, the data in the figure are presented as log2 fold change (log2FC). We screened common differentially expressed miRNAs as target miRNAs for subsequent qRT-PCR validation by constructing a venn diagram (Fig. 3). Among the three miRNAs identified as common across all stages of PCa, hsa-miR-6715b-3p was chosen for validation due to its exceptionally high differential expression (64-fold increase in PCa tissues) and its predicted involvement in critical oncogenic pathways. Future studies will aim to validate the remaining miRNAs to assess their potential as biomarkers or therapeutic targets.

### GO enrichment analysis and KEGG analysis of differential MiRNA source genes

**Fig. 4:**
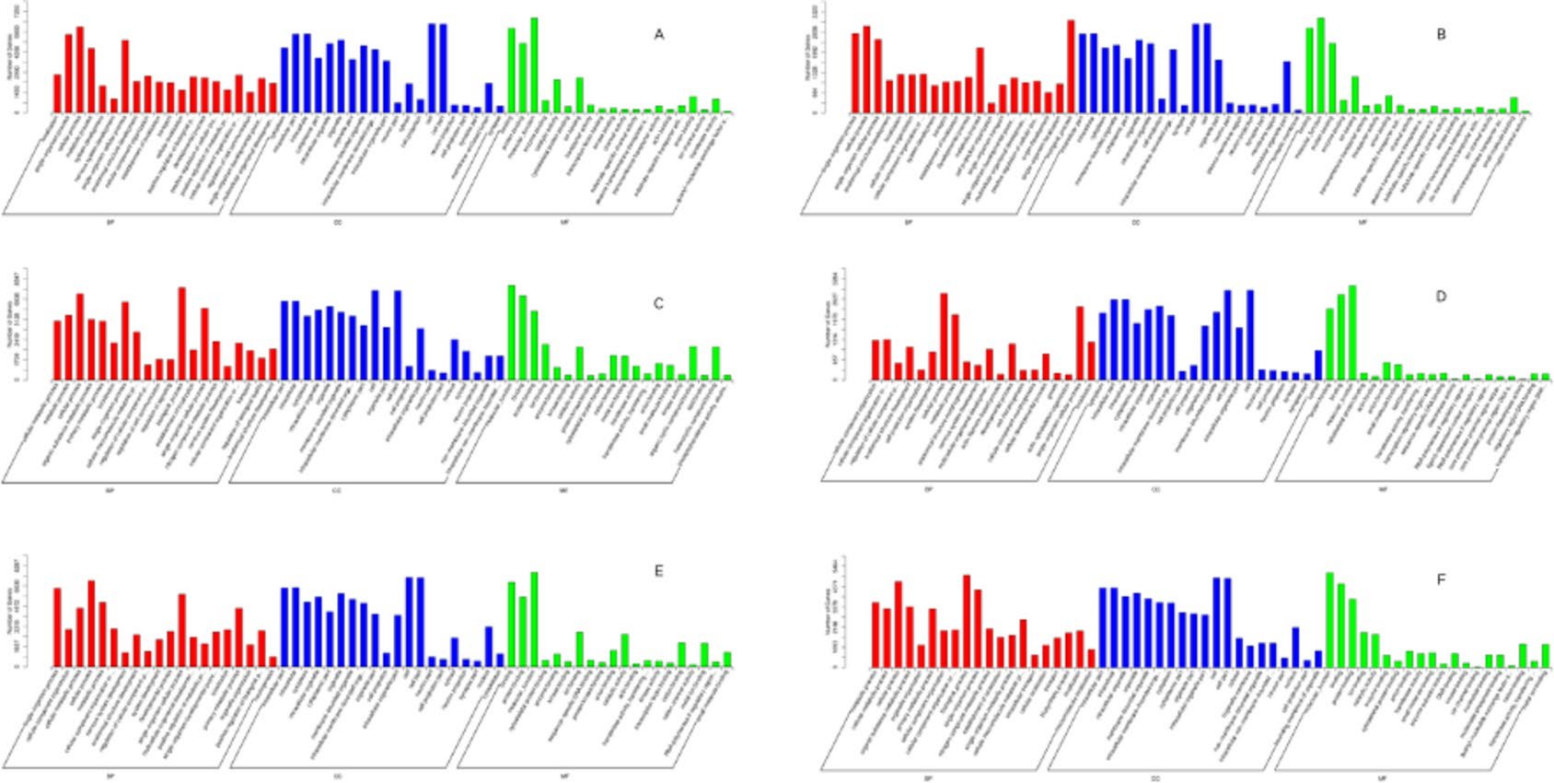
GO enrichment analysis of differentially expressed miRNAs. (**A** early localized PCa VS BPH; **B** local invasion PCa VS BPH; **C** late-stage metastasis PCa VS BPH; **D** local invasion PCa VS early localized PCa; **E** late-stage metastasis PCa VS early localized PCa; **F** late-stage metastasis PCa VS local invasion PCa. Benign prostatic hyperplasia, BPH; Prostate cancer, PCa).

**Fig. 5:**
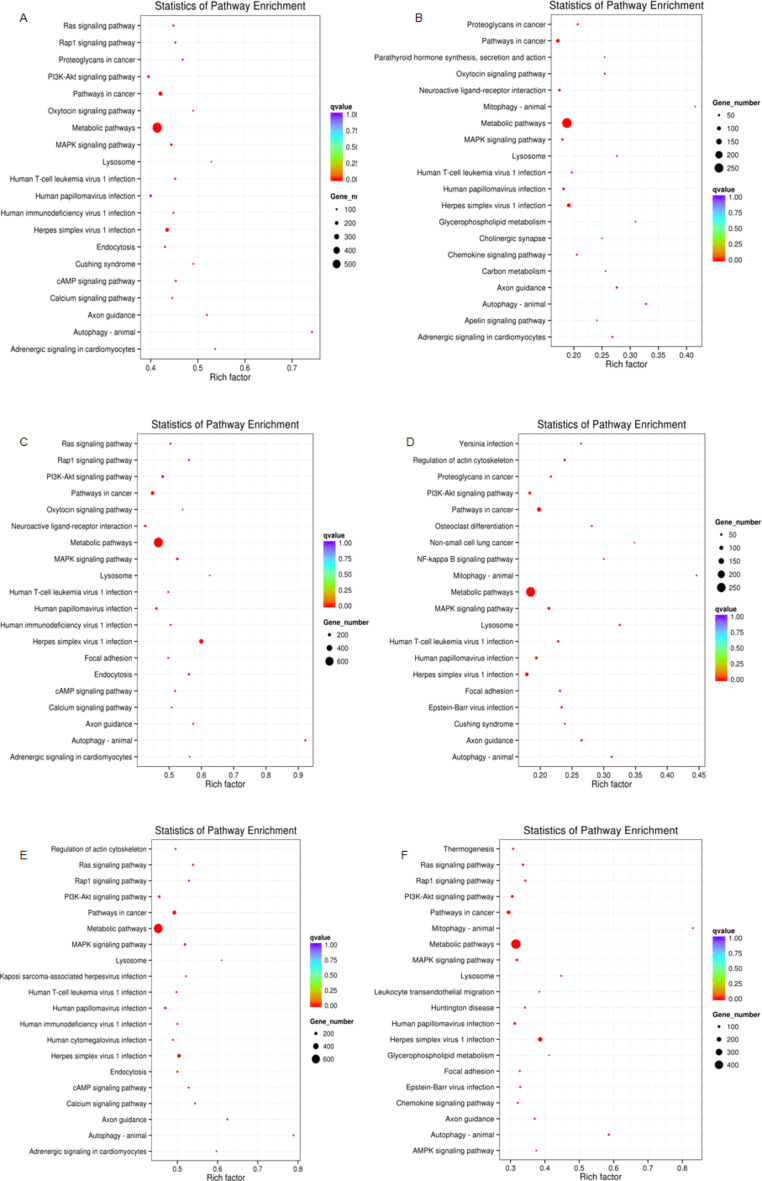
KEGG enrichment analysis of differentially expressed miRNAs. Statistics of pathway enrichment for various signaling pathways based on KEGG. Used with permission from KEGG^33–35^. (**A**) Early localized PCa VS BPH; (**B**) local invasion PCa VS BPH; (**C**) late-stage metastasis PCa VS BPH; (**D**) local invasion PCa VS early localized PCa; (**E**) late-stage metastasis PCa VS early localized PCa; (**F**) late-stage metastasis PCa VS local invasion PCa. (Benign prostatic hyperplasia, BPH; Prostate cancer, PCa).

GO functional enrichment analysis and KEGG analysis were performed on the source genes of differentially expressed miRNAs to indirectly predict the function of miRNAs, and the 20 most significantly enriched maps were selected. GO enrichment analysis (Fig. 4) revealed that the source genes of differentially expressed miRNAs are primarily involved in cellular metabolism, signal transduction, and transcriptional regulation. KEGG analysis further demonstrated that these miRNAs may influence prostate cancer progression through metabolic pathways, the MAPK signaling pathway, and the autophagy pathway. The MAPK signaling pathway plays a crucial role in prostate cancer cell proliferation, apoptosis, and drug resistance, while the autophagy pathway has been shown to have a dual role in prostate cancer cell survival under therapeutic stress. These play an important role in the occurrence, development, metabolism, and transcription of tumors (Fig. 5).

### Target MiRNA prediction

**Fig. 6:**
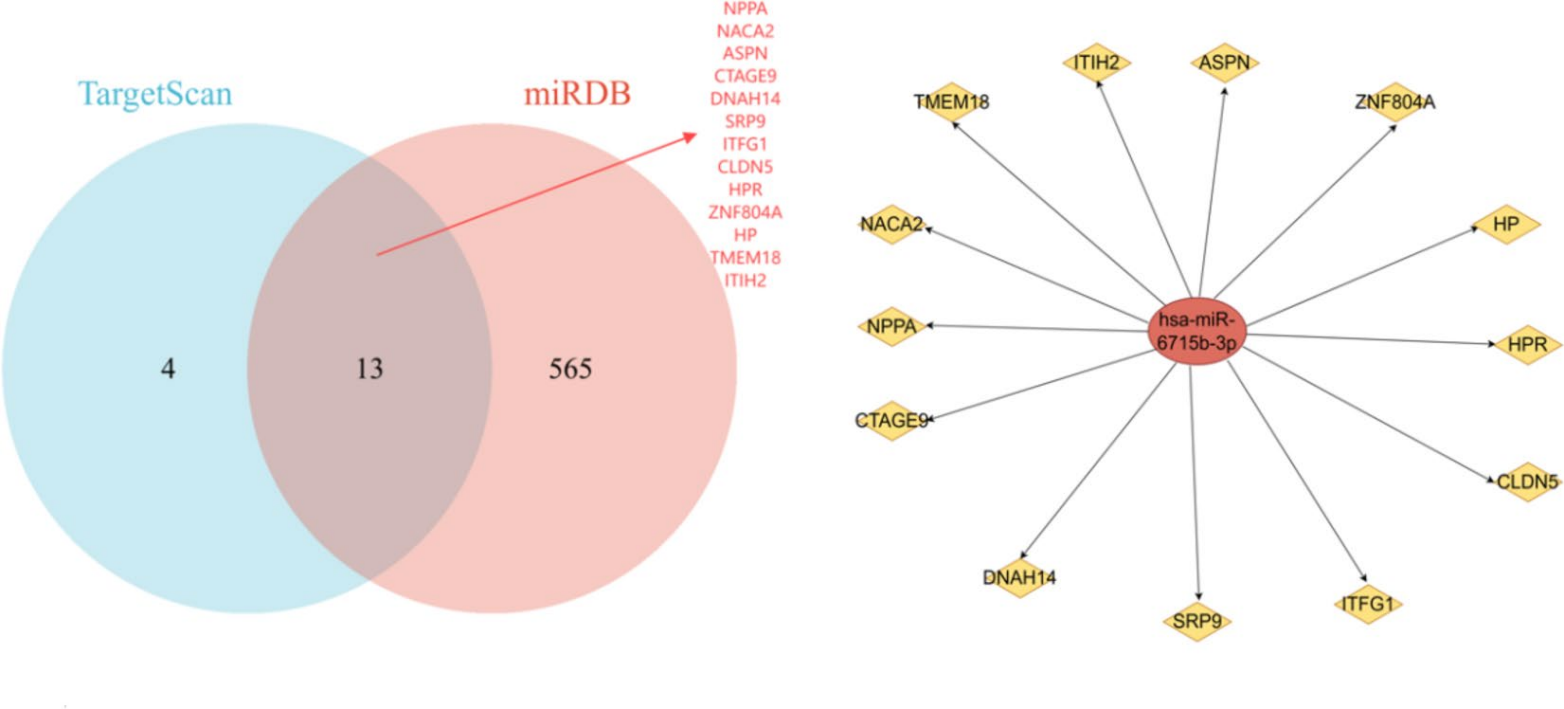
miRNA–mRNA network of miRNA.

MiRNAs can induce gene silencing and downregulate gene expression by binding to mRNAs. We first obtained 578 and 17 target miRNAs of miRNA from the miRDB and TargetScan databases, respectively, and ultimately selected 13 important target mRNAs, which are NPPA, NACA2, ASPN, CTAGE9, DNAH14, SRP9, ITFG1, CLDN5, HPR, ZNF804A, HP, TMEM18, and ITIH2. Then, we constructed a network diagram of AURKB-associated miRNA-mRNA interactions based on the obtained data (Fig. 6).

### Results of qRT-PCR detection

**Fig. 7:**
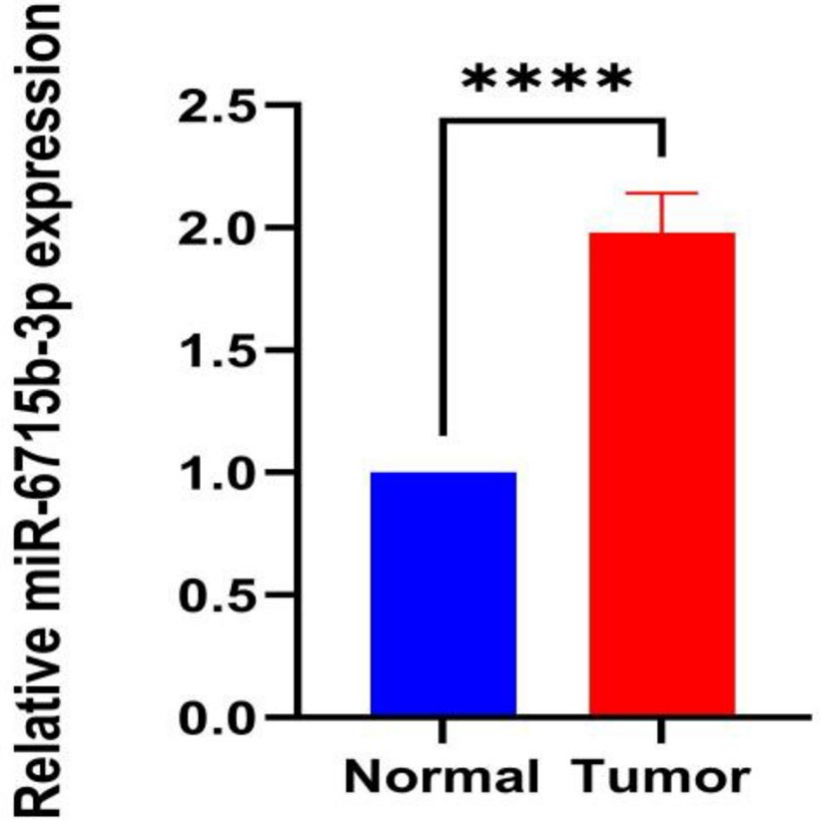
QRT-PCR validation of hsa-miR−6715b−3p expression in PCa and BPH tissues. (Prostate cancer, PCa; Benign prostatic hyperplasia, BPH)

To investigate the expression of hsa-miR-6715b-3p in PCa tissues, we measured the expression levels of hsa-miR-6715b-3p in 10 cases of PCa tissues from different patients and 10 cases of BPH tissues from different patients. The expression level of hsa-miR-6715b-3p was significantly higher in the PCa group than in the BPH group (*P* < 0.001), which is fully consistent with the results of high-throughput sequencing. These results indicate that the findings from high-throughput sequencing are accurate and reliable (Fig. 7).

## Discussion

MiRNAs are approximately 22-nucleotide-long non-coding RNAs that play crucial roles in biological processes such as cell growth, differentiation, and apoptosis by regulating gene expression^10,11^. Dysregulated miRNA expression has been implicated in various cancers, including bladder cancer^12^, breast cancer^13^, renal cell carcinoma^14^, gastric cancer^15^, and lung cancer^16^. In the early screening of prostate cancer, for patients in the PSA gray zone, despite PSA levels being between normal and high-risk ranges, their prostate cancer diagnosis often faces significant uncertainty. Patients in this zone may exhibit complex clinical characteristics, making traditional PSA testing unable to provide accurate diagnostic results^6^. Regarding the diagnostic difficulty of PSA gray zone patients, there may be a potential relationship between APE levels and hsa-miR-6715b-3p, which warrants further exploration. APE (apurinic/apyrimidinic endonuclease), as a DNA repair enzyme, plays a critical role in tumor initiation and progression^17^. Previous studies have indicated that abnormal APE levels may be associated with tumorigenesis, progression, and treatment resistance. hsa-miR-6715b-3p is also believed to be closely related to tumorigenesis in certain cancers by regulating gene expression^18^. Although this study did not specifically investigate the direct relationship between APE and hsa-miR-6715b-3p, based on existing literature, the potential association between the two merits further research, particularly in the context of PSA gray zone patients.

Recent studies highlight the significant role of miRNAs in PCa (PCa), with miR-21 and miR-18a being notable examples. Research by Angel et al. demonstrated that hypoxia is pivotal in the upregulation of miR-21 in PCa, leading to the downregulation of the tumor suppressor gene RHOB, ultimately promoting tumor progression^19^. Another study by Al-Kafaji et al. found significantly elevated levels of miR-18a in the peripheral blood of PCa patients compared to those with BPH (BPH) and healthy individuals, suggesting that miR-18a may serve as a novel, non-invasive biomarker to distinguish PCa from BPH^20^. Therefore, identifying miRNAs involved in PCa is particularly important.

In our study, high-throughput sequencing identified 1,526 miRNAs, from which 1,457 known miRNAs were annotated using the miRBase database, miREvo, and mirdeep2 software. Differentially expressed miRNAs were identified through comparisons between groups, followed by Gene Ontology (GO) enrichment analysis of their source gene sets. GO enrichment analysis indicates that differentially expressed miRNAs are primarily involved in cellular metabolism and transcriptional regulation. However, a deeper interpretation of the Kyoto Encyclopedia of Genes and Genomes (KEGG) pathway analysis suggests that the significant enrichment of the MAPK signaling pathway and autophagy pathway may play a pivotal role in prostate cancer progression. Recent studies have shown that the MAPK signaling pathway can promote prostate cancer progression by regulating androgen receptor (AR) signaling, and its abnormal activation is closely associated with drug resistance. Meanwhile, autophagy plays a complex role in prostate cancer, as it can both support cancer cell survival under nutrient deprivation or therapeutic pressure and, under specific conditions, induce cancer cell apoptosis. For instance, previous studies have indicated that autophagy may influence prostate cancer progression through the AMPK and PI3K/AKT signaling axes^21,22^. These findings suggest that miRNAs may play a key role in regulating these critical signaling pathways, offering potential targets for prostate cancer therapy. It is well established that metabolic processes^23^, the MAPK signaling pathway^24^, and autophagy^25^ play significant roles in cancer development. Liu et al. demonstrated that ANLN regulates the MAPK signaling pathway, stabilizing the oncogene c-Myc and activating this pathway via IGF2BP1 to promote PCa progression^26^. Yuan posited that autophagy has dual roles in PCa, with upstream regulatory mechanisms mainly involving the AMPK and PI3K/AKT signaling pathways. Autophagy encompasses five major stages: initiation, nucleation, maturation, fusion, and degradation, with key autophagy-related proteins serving as regulatory targets at each stage, thus facilitating the modulation of tumor mechanisms^27^. This study provides the first evidence that hsa-miR-6715b-3p is consistently overexpressed across different stages of PCa, supporting its potential as a robust biomarker for disease progression. Our findings align with previous studies demonstrating the role of miRNAs in PCa but extend this knowledge by identifying a novel miRNA that exhibits differential expression from early localized tumors to metastatic PCa. Furthermore, functional enrichment analysis suggests that hsa-miR-6715b-3p may regulate key pathways involved in PCa progression, particularly the MAPK and autophagy pathways, which are known to contribute to androgen receptor signaling, therapy resistance, and tumor cell survival. These findings highlight a previously unrecognized miRNA that may serve as both a biomarker and a potential therapeutic target. Future studies should focus on the functional characterization of hsa-miR-6715b-3p and its downstream targets to determine its role in PCa progression and treatment response. Our study lays the groundwork for the integration of miRNA-based biomarkers into PCa diagnostics and precision medicine approaches.

We validated the expression of miR-6715b-3p using qRT-PCR. The results indicated that miR-6715b-3p was significantly overexpressed in PCa tissues compared to BPH, reinforcing the reliability of our findings. miR-6715b-3p, a novel and annotated miRNA, has been under-researched in the context of current diseases. Prior studies identified miR-6715b-3p as essential for regulating autophagy through SESN1 in Huntington’s disease^28^. In cancer research, its relevance was noted in a study by Ahmad et al. on colorectal cancer, where miR-6715b-3p was the most significantly upregulated miRNA, with a new Siglec-15-Sia axis inhibitor targeting this miRNA and oncogenes to induce colorectal cancer cell death, suggesting its pro-tumor role^29^. This aligns with our qRT-PCR findings of elevated miR-6715b-3p levels in PCa tissues, laying the groundwork for future research.

In this study, we identified miR-6715b-3p as a potential biomarker in prostate cancer. Through bioinformatics analysis and literature review, miR-6715b-3p was found to potentially target several genes associated with the initiation and progression of prostate cancer, particularly PTEN (phosphatase and tensin homolog), which plays a critical role as a tumor suppressor in the regulation of prostate cancer. Downregulation of PTEN activates the PI3K/Akt signaling pathway, promoting cell proliferation, survival, and anti-apoptotic effects, which is especially significant in prostate cancer cells^30^. Previous studies have shown that miR-6715b-3p binds to the 3’ untranslated region (UTR) of PTEN, inhibiting its translation or promoting mRNA degradation, thereby reducing PTEN protein expression^31^. This is closely related to the proliferation and metastasis of prostate cancer cells.

Although this study did not directly investigate the relationship between miR-6715b-3p and other potential target genes (such as VEGF, EGFR, etc.), existing studies suggest that these genes may also be targets of miR-6715b-3p and play important roles in prostate cancer metastasis, angiogenesis, and immune evasion^32^. Therefore, future research could further explore the interactions between miR-6715b-3p and other key genes, as well as their impact on the prostate cancer microenvironment.

Our study leverages current high-throughput sequencing techniques to analyze miRNA expression profiles in prostate tissues from PCa and BPH patients across various clinical stages. We conducted preliminary functional predictions and analyses of differentially expressed miRNAs and validated the novel miRNA miR-6715b-3p, providing new targets for studying the molecular mechanisms underlying PCa. However, limitations include the small sample size for high-throughput sequencing, which restricts the clinical data available for correlational analysis with clinical parameters and staging. Additionally, some identified miRNAs are not currently cataloged or formally named in existing databases, precluding further study. Lastly, we verified only the differential expression of miR-6715b-3p between PCa and BPH without delving into its specific molecular regulation in PCa.

## Conclusion

This study presents a comprehensive analysis of miRNA expression in prostate cancer across different clinical stages and identifies hsa-miR-6715b-3p as a promising novel biomarker for disease progression. Our findings highlight its potential role in critical oncogenic pathways and provide a basis for further functional studies to explore its clinical utility. Given its significant overexpression in PCa tissues and its potential role in oncogenic pathways, further functional studies are warranted to determine its precise molecular mechanism and clinical utility. Additionally, future research should validate other differentially expressed miRNAs identified in this study and explore their roles in therapy resistance and disease progression. These findings contribute to the expanding knowledge of miRNA-based biomarkers and provide potential targets for precision medicine approaches in prostate cancer.

## Electronic supplementary material

Below is the link to the electronic supplementary material.

Supplementary Material 1

Supplementary Material 2

## Data Availability

The data generated during this study is available from the corresponding author on reasonable request. The miRNA data supporting the findings of this study have been deposited in the NCBI BioProject database (accession PRJNA1226508). To review BioProject accession PRJNA1226508: Go to http://www.ncbi.nlm.nih.gov/bioproject/1226508.
