# Supplementary material for: MiRNA expression profiling and clinical implications in prostate cancer across various stages
Source: Sci Rep. 2025 Mar 5;15:7771. doi: 10.1038/s41598-025-92091-9 (PMC11882840; doi:10.1038/s41598-025-92091-9)
Supplement: Supplementary file 1 — Supplementary Material 1 [file 41598_2025_92091_MOESM1_ESM.docx]

**Supplementary documents**

**Table 1 Statistics on the number of differentially expressed miRNAs between groups**

| GROUP | DIFF | UP | DOWN |
| --- | --- | --- | --- |
| Localised invasion vs prostate enlargement | 21 | 14 | 7 |
| Localised invasion vs. early confinement | 11 | 7 | 4 |
| late-stage metastatic tumors vs. local invasion | 34 | 7 | 27 |
| late-stage metastatic tumors vs prostate enlargement | 42 | 23 | 19 |
| late-stage metastatic tumors vs. early confinement | 87 | 36 | 51 |
| Early confined vs prostate enlargement | 33 | 13 | 20 |

**Table 2 Results of differential analysis of miRNA expression (partial)**

| **clusters** | **UP** | **DOWN** |
| --- | --- | --- |
| **local invasion vs prostate enlargement** | novel_417  hsa-miR-141-3p  hsa-miR-182-3p  hsa-miR-96-5p  hsa-miR-7-1-3p | hsa-miR-4433a-3p  hsa-miR-4433b-5p  hsa-miR-205-5p  hsa-miR-184  hsa-miR-4521 |
| **Localised metastasis vs early confinement** | hsa-miR-200b-5p  hsa-miR-486-3p  hsa-miR-486-5p  hsa-miR-92a-1-5p  hsa-miR-874-3p | hsa-miR-133a-5p  hsa-miR-222-5p  hsa-miR-377-3p  hsa-miR-381-3p  hsa-miR-205-3p |
| **late-stage metastatic tumors vs local invasion** | hsa-miR-146a-5p  hsa-miR-1273h-5p  hsa-miR-18a-3p  hsa-miR-206  hsa-miR-625-5p | hsa-miR-490-5p  hsa-miR-135a-5p  hsa-miR-490-3p  hsa-miR-106a-3p  hsa-miR-141-3p |
| **late-stage metastatic tumors vs. prostate enlargement** | hsa-miR-18a-3p  hsa-miR-346  hsa-let-7d-3p  hsa-miR-625-3p  hsa-miR-210-5p | hsa-miR-4433a-3p  hsa-miR-4433b-5p  hsa-miR-184  hsa-miR-218-5p  hsa-miR-136-5p |
| **late-stage metastatic tumors vs. early confinement** | hsa-miR-150-5p  hsa-miR-18a-3p  hsa-miR-4521  hsa-miR-92a-1-5p  hsa-miR-92b-3p | hsa-miR-135a-5p  hsa-miR-34c-5p  hsa-miR-374a-5p  hsa-miR-377-3p  hsa-miR-582-5p |
| **Early confined vs prostate enlargement** | hsa-miR-377-3p  hsa-miR-425-5p  hsa-miR-449c-5p  hsa-miR-542-3p  hsa-miR-6715b-3p | hsa-miR-200b-3p  hsa-miR-200b-5p  hsa-miR-4433a-3p  hsa-miR-4433b-5p  hsa-miR-4521 |
